# Cost-effectiveness of herpes zoster vaccination in patients with rheumatoid arthritis

**DOI:** 10.1093/rheumatology/keag448

**Published:** 2026-08-19

**Authors:** Gunhild Hagen, Anna K Opheim, Joakim Overbo, Eline Aas, Sella A Provan

**Affiliations:** Research Center for Treatment of Rheumatic and Musculoskeletal Diseases (REMEDY), Diakonhjemmet Hospital, Oslo, Norway; Cluster for Health Services Research, Norwegian Institute of Public Health, Oslo, Norway; Department of Health Economics and Health Management, University of Oslo, Oslo, Norway; Department of Virology, Norwegian Institute of Public Health, Oslo, Norway; Cluster for Health Services Research, Norwegian Institute of Public Health, Oslo, Norway; Department of Health Economics and Health Management, University of Oslo, Oslo, Norway; Research Center for Treatment of Rheumatic and Musculoskeletal Diseases (REMEDY), Diakonhjemmet Hospital, Oslo, Norway; Section for Public Health, University of Inland Norway, Elverum, Norway

**Keywords:** rheumatoid arthritis, herpes zoster, vaccine, cost-effectiveness, Markov model

## Abstract

**Objectives:**

To assess the cost-effectiveness of vaccinating patients with rheumatoid arthritis (RA) with the adjuvanted recombinant zoster vaccine (RZV), compared with no vaccination.

**Methods:**

To predict health gains and costs from vaccination, we developed a decision-analytic Markov model with five health states: no herpes zoster, herpes zoster (HZ), postherpetic neuralgia (PHN), recovered from HZ and death. The model follows a hypothetical cohort of 10 000 patients with RA over a lifetime perspective and records events of HZ and PHN, along with quality-adjusted life years (QALYs) and costs from a healthcare perspective. Costs and health effects were discounted with 4% annually. Model inputs were collected from published literature and publicly available sources. Cost-effectiveness was evaluated using the incremental cost-effectiveness ratio (ICER), expressed as EUR per QALY, compared with a cost-effectiveness threshold of EUR 23 650 per QALY. We performed one-way and probabilistic sensitivity analyses.

**Results:**

Vaccination led to a reduction of 2550 cases of HZ and 1303 cases of PHN per 10 000 patients with RA vaccinated, resulting in an ICER of EUR 11 205/QALY. One-way sensitivity analyses revealed that the results were particularly sensitive to changes in the probability of developing PHN as a sequela of HZ. The probabilistic analysis indicated that the RZV had a 98% probability of being cost-effective at the assumed threshold value.

**Conclusion:**

Vaccinating patients with RA using RZV results in better health at higher cost compared with no vaccination. Under the assumptions made, vaccination is likely to be considered cost-effective for patients with RA.

Rheumatology key messages

- Patients with rheumatoid arthritis have an increased risk of herpes zoster and associated complications.
- Herpes zoster vaccination appears cost-effective for patients with rheumatoid arthritis aged 50 years and older.
- Findings support integrating herpes zoster vaccination from age 50 into clinical guidelines and health policies.

## Introduction

Due to significant clinical progress, patients with rheumatoid arthritis (RA) have several pharmacological treatment options, including glucocorticoids, methotrexate and inhibitors of tumour necrosis factor (TNF), Janus kinase (JAK) and interleukin 6 [1]. While these treatments are highly effective in controlling disease activity and symptoms, they suppress the immune system, increasing the patient’s susceptibility to infections [2]. Herpes zoster (HZ) is caused by a reactivation of varicella virus, which may lie dormant in the body following a childhood infection. A reactivation can typically be caused following a weakening of the immune system, resulting from age or from immunosuppressive therapies. The risk increase varies across age, ethnicity and the medications used to treat RA. It is generally two to three times higher than in the general population [3]. Patients with RA also tend to experience more severe infections [4].

HZ presents as a painful, blistering rash that typically endures for 2–4 weeks. The pain is caused by inflammation in the affected nerve, and fluid-filled blisters develop along the corresponding dermatome. The most prevalent complication of HZ is PHN, which is characterized by persistent pain following the resolution of the rash. The pain associated with PHN can be debilitating, significantly impacting various aspects of an individual’s life, including sleep and daily activities. Patients frequently experience weight loss, depression and chronic fatigue in response to this neuropathic pain [5]. Other neurological complications can occur and may include ocular scarring, as well as encephalitis, myelitis and meningitis [6]. However, these complications are less frequent.

Vaccination can effectively prevent HZ. Previously, there were concerns that vaccination of immunocompromised individuals could lead to either opportunistic infections or trigger disease flares. The first available HZ vaccines were live attenuated and should not be used during active immunosuppression [7]. Furthermore, it was uncertain whether immunocompromised individuals could mount an adequate immune response. These concerns have been addressed by new studies and an adjuvanted recombinant zoster vaccine has been approved for immunosuppressed individuals. As a result, vaccination has been shown to be safe and effective in patients with RA, although effectiveness may be somewhat lower than in the general population [8–10].

However, vaccine uptake among patients with RA remains low, with reported coverage rates for HZ as low as 2% [11, 12]. Cost-effectiveness analyses are often used to inform reimbursement decisions and uptake of vaccines in health insurance schemes [13, 14] and the lack of such analyses of HZ vaccination for patients with RA has been cited as one reason why vaccine uptake remains low [15, 16]. To date, no cost-effectiveness analyses of zoster vaccination of patients with RA can be found in the published literature. To fill this evidence gap, we have evaluated the cost-effectiveness of zoster vaccination of patients with RA in Norway.

## Methods

### Analytical overview

We evaluated the cost-effectiveness of the recombinant zoster vaccine for preventing HZ in patients aged 50 years with RA. The model follows a hypothetical cohort of patients with RA over a lifetime perspective and records events of HZ and PHN, along with quality-adjusted life years (QALYs) and costs from a healthcare perspective. Costs and health effects were discounted with 4% per annum. The indicator of cost-effectiveness is the incremental cost-effectiveness ratio (ICER), expressed as EUR per QALY gained. Vaccination is deemed cost-effective if the ICER is below a cost-effectiveness threshold of EUR 23 650 per QALY [17]. The threshold represents the willingness to pay for one QALY. It is sometimes estimated from health opportunity costs and varies across jurisdictions [18]. In Norway, thresholds are higher for more severe conditions [17]; the threshold applied here reflects the relatively limited severity and duration of HZ in most cases.

### Model structure

**Figure 1:**
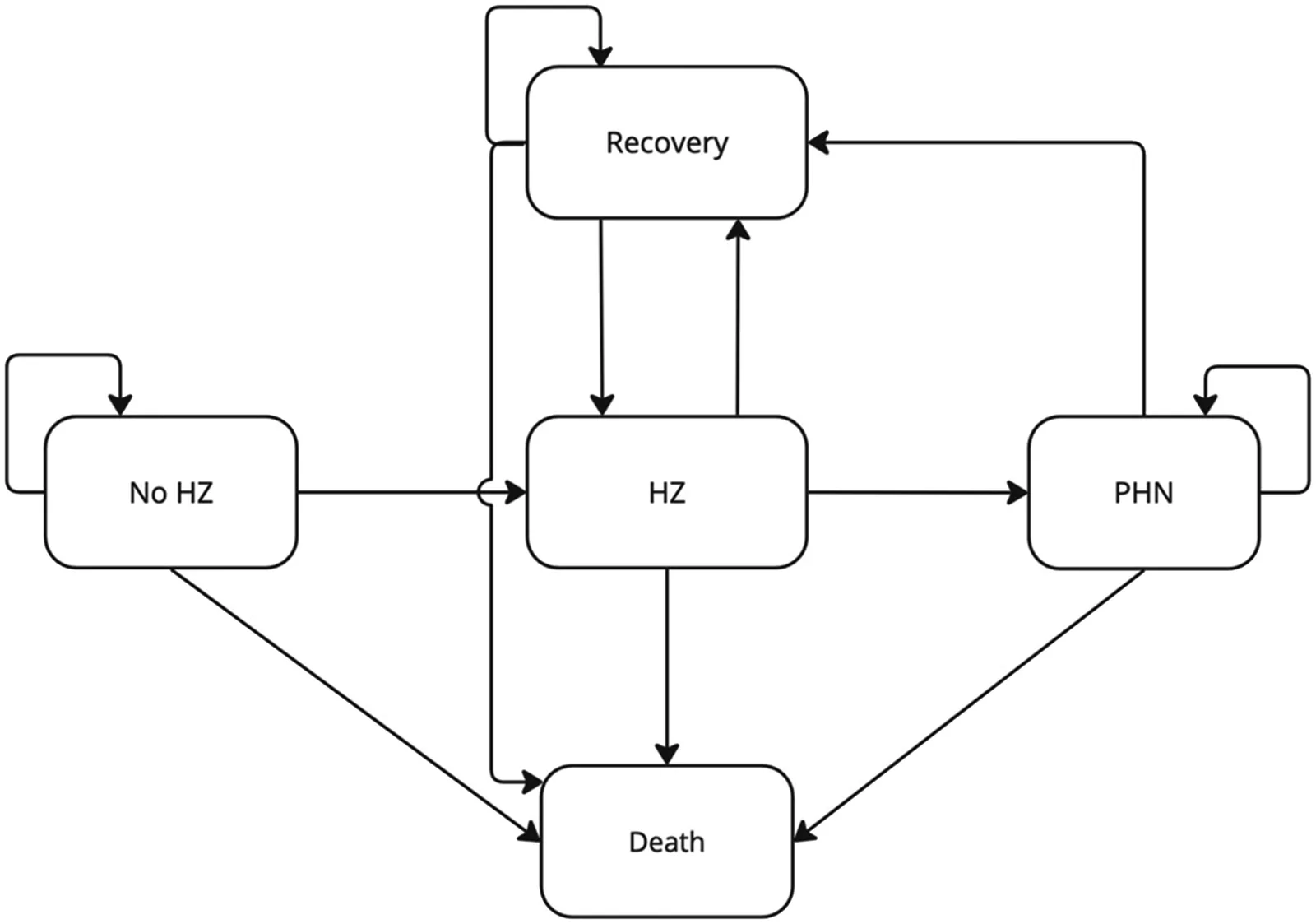
Model structure

We developed a static Markov model with 3-month cycles to simulate a cohort of patients with RA from age 50 years old and until death (Fig. 1). We chose a 3-month cycle length to reflect that PHN is defined as persistent pain 90 days after rash onset [19]. The model included five mutually exclusive health states: *no HZ*, *HZ*, *recovered*, *PHN* and *death*. Modelled patients enter the model in *no HZ*. From *no HZ*, the individuals can remain in *no HZ*, transition to *HZ* or *death*. HZ infection is limited to only one cycle to reflect the 2-to-4-week timeline of the disease. After HZ infection, patients can either move on to *recovered*, *PHN* or *death*. From *PHN*, the individuals can stay, move to *recovered* or *death*. The model allowed patients to stay multiple cycles in the *PHN* state, as over 30% of patients experience persistent pain beyond 12 months [19]. The *recovered* state was included to allow the risk of a second HZ infection to be lower than the risk of primary infection (i.e. 9.1%), as reported in epidemiological data [20].

### Model input parameters

As customary for model-based analyses, input data were obtained from various published sources [21] (Table 1). Following the approach described by Coyle *et al.* [32], we followed a traditional hierarchy of evidence for estimates of clinical effectiveness, while transferability to local context was prioritized for epidemiological (rates of events) and cost parameters.

**Table 1:** Model inputs.

Parameter	Mean value	Distribution used for probabilistic analysis	Parameters used to inform distribution	Reference
**Epidemiology**				
HZ^a^ incidence rate in patients with RA per 1000 patient years, age 50–64 years, 65+	17.3, 27			Södergren *et al.* [20]
Mortality rate per 100 000, patient age 50–59, 60–69, 70–79, 80+	0.08, 0.19, 0.71, 8.13			Statistics Norway
Annual waning of vaccine effect per annum %	2.50	Beta	24.4/949.65	Linear regression based on Strezova *et al.* [25]
Uptake of vaccination in Patients with RA^b^ in%	60	Fixed		Nakafero *et al.* [28]
Probability of developing post-herpetic neuralgia in %	25	Beta	7.7/23	Domínguez-Casas *et al.* [22]
Probability of hospitalization following zoster infection, age 50–69, 70–79, 80+ ,%	2.72,3.55,4.96	Beta	24.3/868.8, 24,1/654.1, 23.7/454.3	Mirinaviciute *et al.* [31]
**Clinical effectiveness of vaccination in %**				
HZ^a^ infection, age 50–69, 70+	93, 84	Beta	3/0.2	Dagnew *et al.* [36]
Post-herpetic neuralgia	90	Beta	25.3/2.8	Cunningham *et al.* [35]
**Costs (EUR)**				
Price per dosage of vaccine	163	Gamma		List price per 31/03/2025
Administration cost	22.5	Gamma	25/1	Norwegian Directorate of Medicinal Products [30]
Visits to general practitioner	32	Gamma	25/1	Norwegian Directorate of Medicinal Products [30]
Hospitalization	2732	Gamma	25/109	Norwegian Directorate of Health 2024
Medications	47	Gamma	25/2	Assumed treatment with antiviral medication, based on official list price of valaciclovir
Medication postherpetic neuralgia	72	Gamma	25/3	Assumed treatment with capsaicin 0.075% cream
**Health-related quality of life**				
Baseline utility values Patients with RA,^b^ age 50–69, 70+	0.725, 0.719	Beta	7.08/2.69	Linde *et al.* [27]
Herpes zoster (patients that does not develop postherpetic neuralgia), age 50–69, 79+	0.692, 0.671	Beta	5.5/2.7	Lui *et al.* [23]
Herpes zoster (patients that develop postherpetic neuralgia), age 50–69, 79+	0.38, 0.33	Beta	17/28.3, 12.6/24.84	Lui *et al.* [23]
Utility for postherpetic neuralgia, age 50–69, 70+	0.48, 0.37	Beta	11.27/19.05	Lui *et al.* [23]

a Herpes zoster.

b Rheumatoid arthritis.

#### Epidemiological parameters

We obtained HZ incidence rates in patients with RA from a study by Södergren *et al.*, who reported data from a regional population-based register study in Sweden. The study included 9828 persons with RA, where 1036 cases of HZ were identified over the period 2014–19 [20]. Based on these findings, the incidence rates were 17.3 and 27 per 1000 patient years for the age groups 50–65 and 65+, respectively. All-cause mortality rates were obtained from Statistics Norway. Since recent research indicates no increased mortality in patients with RA on modern treatments, no additional mortality risk was added [33]. Incidence of HZ and all-cause mortality were modelled as being age-dependent to reflect observed disease patterns. The probability of developing PHN was assumed to be 25% in the base case [22]. This was based on a prospective cohort study, including 392 patients (mean age 59 ± 13 years; 78.8% female) with RA enrolled in a hospital vaccination programme and followed longitudinally for a mean of 137 ± 110 months. Patients were treated with glucocorticoids (63.3%), conventional synthetic DMARDs (50%), biologic DMARDs (50%) and a minority JAK inhibitor.

#### Vaccine effectiveness

Randomized trials of the recombinant zoster vaccine have shown an overall efficacy of 97% for preventing HZ [34]. In older adults the efficacy is somewhat lower at 90% [35]. Because patients with RA may generate a weaker immune response to vaccination, we assumed vaccine effectiveness as reported in post-hoc analyses of patients with immune-mediated diseases included in the randomized controlled trials, i.e. a protection against HZ of 93% and 84%, in patients below and over 70 years olds respectively [36]. The protection against PHN was not reported specifically for this subpopulation; hence we assumed an overall effect on this outcome of 90%.

Vaccine effectiveness was assumed to decline gradually, with an annual waning rate of 2.5%. Waning was estimated using a linear regression of the data presented by Strezova *et al.* [25]. Based on current evidence, we did not include booster doses, nor any serious adverse events from vaccination [37]. In the base case analysis, we assumed that all individuals completed the full two-dose vaccination schedule. Vaccine uptake was set to 60%, based on uptake reported for pneumococcal vaccination in patients with immune mediated inflammatory diseases [28].

#### Health-related quality of life

Baseline utility values for RA and utility values for HZ infection and PHN were obtained from published literature [23, 27]. We adjusted the values for HZ infection and PHN to the RA population using the multiplicative method [24]. Utility values during the HZ infection varied depending on whether patients went on to develop PHN. The patients who only experienced one cycle of HZ and then recovered had a higher utility during HZ than patients who would go on to have PHN. This reflects the patterns of utility observed in the two groups, where the development of PHN often can be predicted by higher pain levels in early stages of infection.

#### Costs

For this analysis, we converted Norwegian kroner to euros using a conversion of 1 NOK = 0.086 EUR. The vaccine price (EUR 165 per injection) was obtained from the Norwegian Medical Products Agency on 5 March 2025, and administration costs were assumed to be EUR 23 per injection [30].

Patients with HZ were assumed to visit their general practitioner, with an average of 1.92 consultations per HZ episode [31]. The cost of antiviral treatment was EUR 47 per episode, and each visit to the general practitioner EUR 32. In 4% of cases, infection resulted in hospitalization with an average cost of EUR 2732 [31]. Hospitalization costs were estimated by applying diagnosis-related group weights for ICD-10 codes B020-B029 [31]. For patients with PHN, an additional cost incurred in the subsequent cycles, reflecting prolonged pain management, i.e. treatment with capsaicin cream, estimated at EUR 72.

#### Sensitivity analyses

We performed one-way sensitivity analyses to explore how changes in individual parameter values affected the results. One-way sensitivity analyses were presented in a tornado diagram, showing the impact of changing parameters from a lower to a higher value on the ICER [38]. In the tornado diagram, input parameters are ranked according to their impact on the ICER, with the most impactful parameters on top and then descending. As recommended in methods guidelines, lower and higher values were chosen to reflect the uncertainty in the underlying evidence base [38]. For vaccine effectiveness on rate of infection, we assumed a lower value of 69% based on real-world data reported by Rayens *et al.* [8], while the high value of 98% is the overall effectiveness reported in the randomized controlled trials [34]. For vaccine protection against PHN, we assumed the upper and lower end of the 95% confidence interval reported from trials, i.e. a lower value of 70% and a higher of 97% [35].

For the estimate of the percentage of infected patients developing post-herpetic neuralgia the lower value was set to be 2% and the higher value 35%. This was based on the studies by Kawai *et al.* [19] who report a range from 5% to 35% while Giannelos *et al.* report a range from 2% to 35% [29]. For uptake of vaccination in patients with RA, we assumed a lower value of 5% reported by Wong *et al.* [12] and a higher value of 95%, the uptake seen in the Norwegian childhood vaccination programme. For the incidence rate, the upper and lower part of the 95% confidence interval around the point estimates in Södergren was used. Incidence rates vary geographically, with age and sex and increases over time [39]; this range reflects the uncertainty for our population (Norwegian Patients with RA), while the range reported in the literature is much wider. For vaccine waning, we assumed a lower value of 1% per annum and a higher valued of 5%. Other parameters, such as vaccine price, cost of hospitalization and others, were varied based on assumptions, as we had no evidence for range, but still wanted to check sensitivity. Additionally, we included a separate threshold analysis on the probability of developing PHN and a scenario analysis with real-world effectiveness as reported by Rayens *et al.* [8]. As 75.4% of patients completed both doses in this study, we also accounted for this in the cost of the vaccine.

To examine the combined impact of parameter uncertainty on the result, we conducted a probabilistic sensitivity analysis, assigning probability distributions to each parameter following the approach described by Briggs *et al.* [26]. We performed 10 000 Monte Carlo simulations, plotted the results on the cost-effectiveness plane and created cost-effectiveness acceptability curves (CEACs), presenting the likelihood of HZ vaccine to be cost-effective over a range of different cost-effectiveness thresholds.

#### Software

All analyses were conducted in Microsoft Excel.

### Patient and public involvement statement

Patients and public representatives were not involved in the modelling part of this project but will be involved in disseminating results after publication. The Research Center for Treatment of Rheumatic and Musculoskeletal Diseases (REMEDY) at Diakonhjemmet hospital has its own patient council User participation in research, who will assist us in dissemination. As the study included no patient level data, ethical approval was not required.

## Results

Vaccinating a cohort of 10 000 Patients with RA led to an estimated reduction of 2550 cases of HZ and a reduction of 1303 cases of PHN. This reduced disease burden translated into 0.016 QALYs gained per eligible patient and an additional cost of EUR 185. Together these effects resulted in an ICER of EUR 11 205 QALY gained (Table 2).

**Table 2:** Base case results, QALYs and costs discounted and half-cycle corrected.

Base case	RZV^a^	No vaccine	Difference
**Number of cases per cohort of 10 000 eligible persons**			
Total cases HZ^b^	3621	6171	2550
Total cases PHN^c^	240	1543	1303
**Costs (€) and QALYs^d^ per eligible person**			
Total costs	265.38	80.7	184.68
Vaccination costs	225.33	–	225.33
Primary care costs	10.87	19.83	−8.96
Hospital costs	15.53	27.44	−11.91
QALYs^d^	13.25	13.27	0.016
**ICER (€ per QALY): 11 205**			

a Recombinant zoster vaccine.

b Herpes zoster.

c Postherpetic neuralgia.

d Quality-adjusted life years.

**Figure 2:**
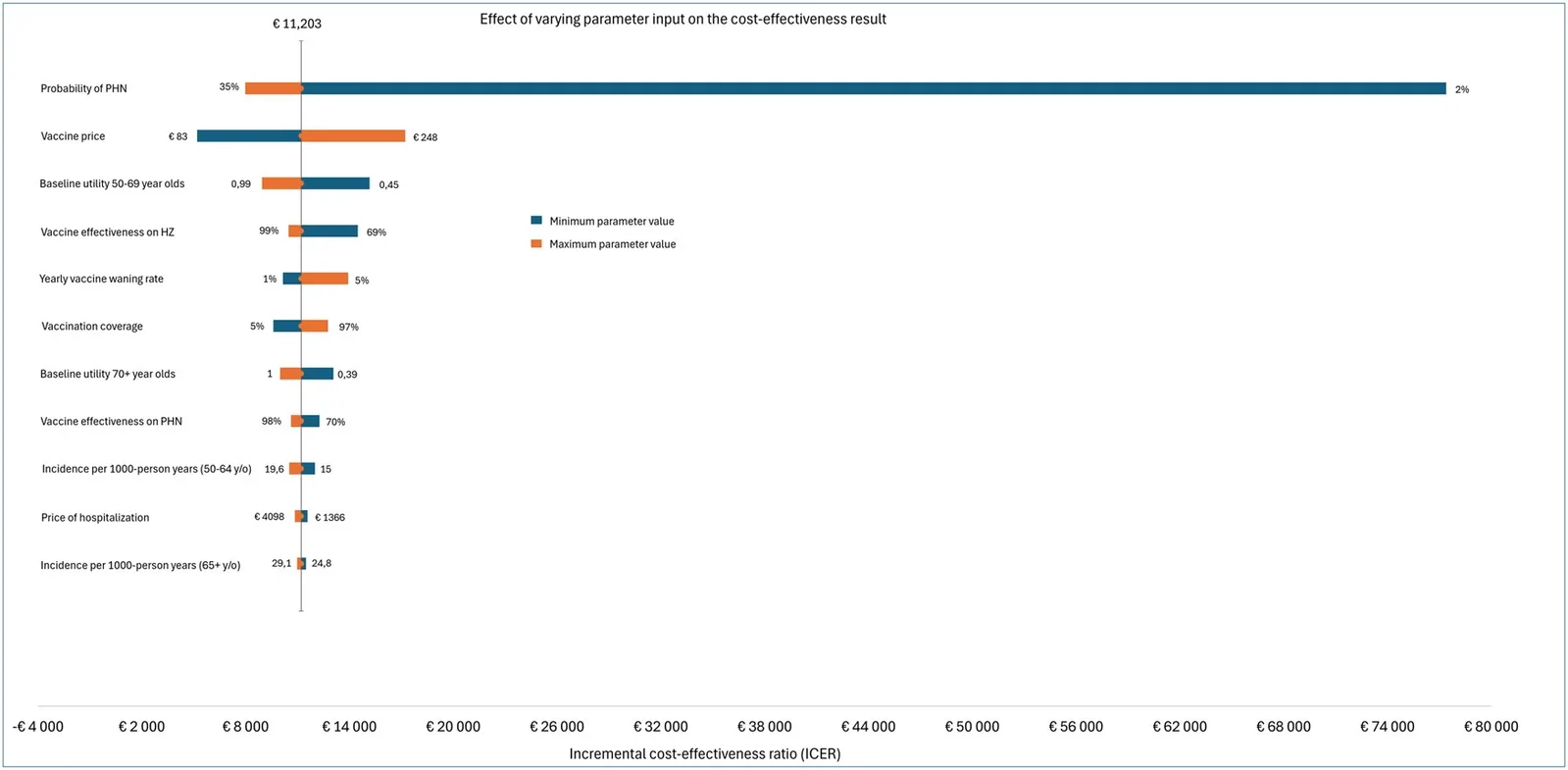
Tornado diagram displaying the sensitivity of the ICER to changes in input data

**Figure 3:**
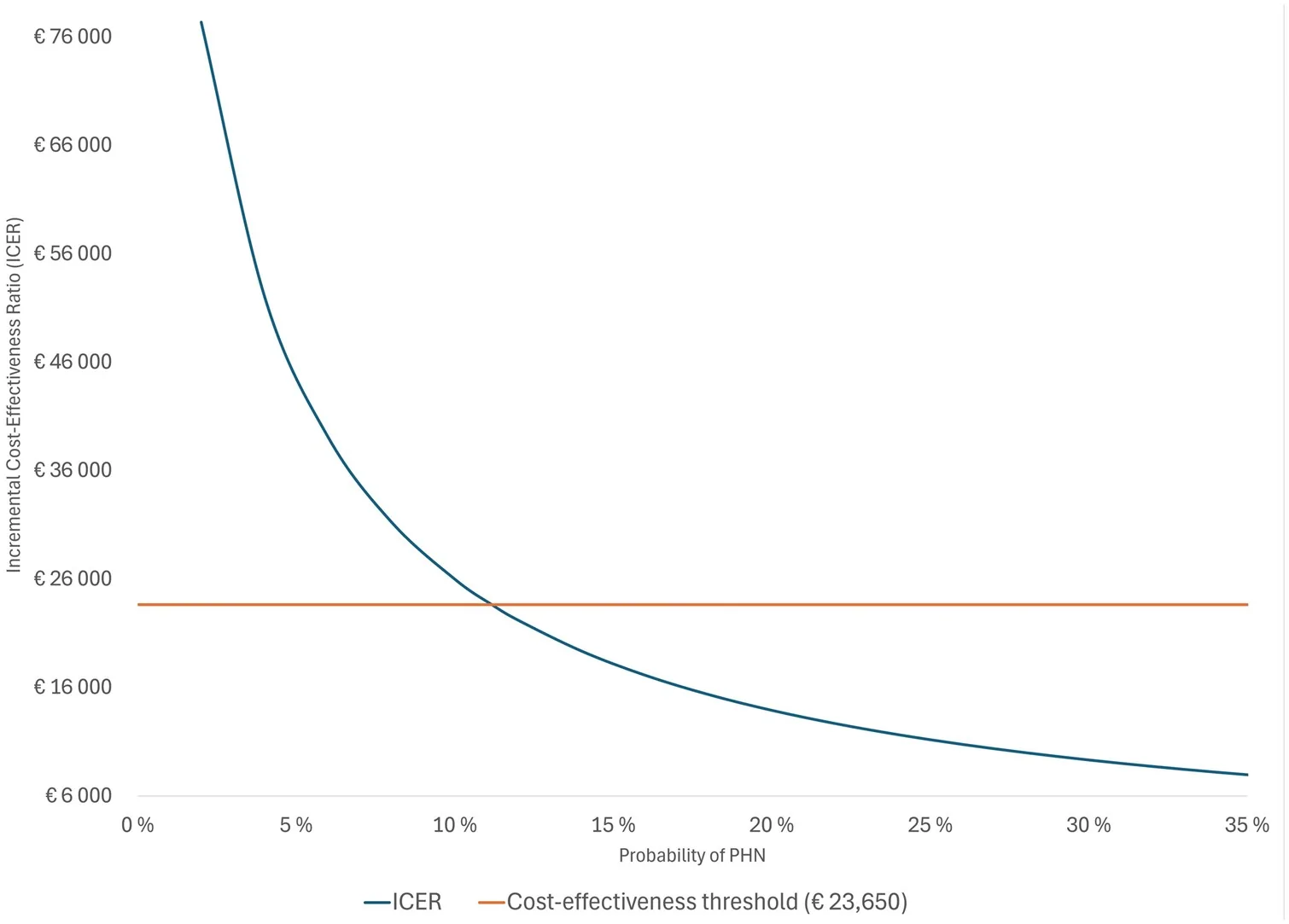
One-way sensitivity analysis on the effect of probability of post-herpetic neuralgia on the estimated incremental cost-effectiveness ratio (ICER)

The tornado diagram revealed that the ICER was particularly sensitive to the probability of developing PHN. A lower probability of 2% increased the ICER to EUR 90 321 per QALY, while a higher probability of 35% reduced the ICER to EUR 11 450 per QALY (Fig. 2). The ICER was also sensitive to vaccine effectiveness, baseline health-related quality of life and vaccine price. When we assumed a vaccine effectiveness as reported in a real-world setting, the ICER increased to EUR 14 485 per QALY. Only changes in the probability of developing PHN changed the ICER to a degree that would bring the ICER above the assumed threshold value of EUR 23 650 per QALY. In the threshold analysis on the probability of developing PHN, we find that vaccination is cost-effective at the assumed threshold if the probability of developing PHN is above 11% (Fig. 3). At a higher cost-effectiveness threshold, vaccination would be cost-effective at a lower probability. When we assumed real-world effectiveness and a 75.4% completion of the second dose, this resulted in an incremental cost of €167 and an incremental QALY gain of 0.013 per eligible patient, giving an ICER of €12 543 per QALY.

**Figure 4:**
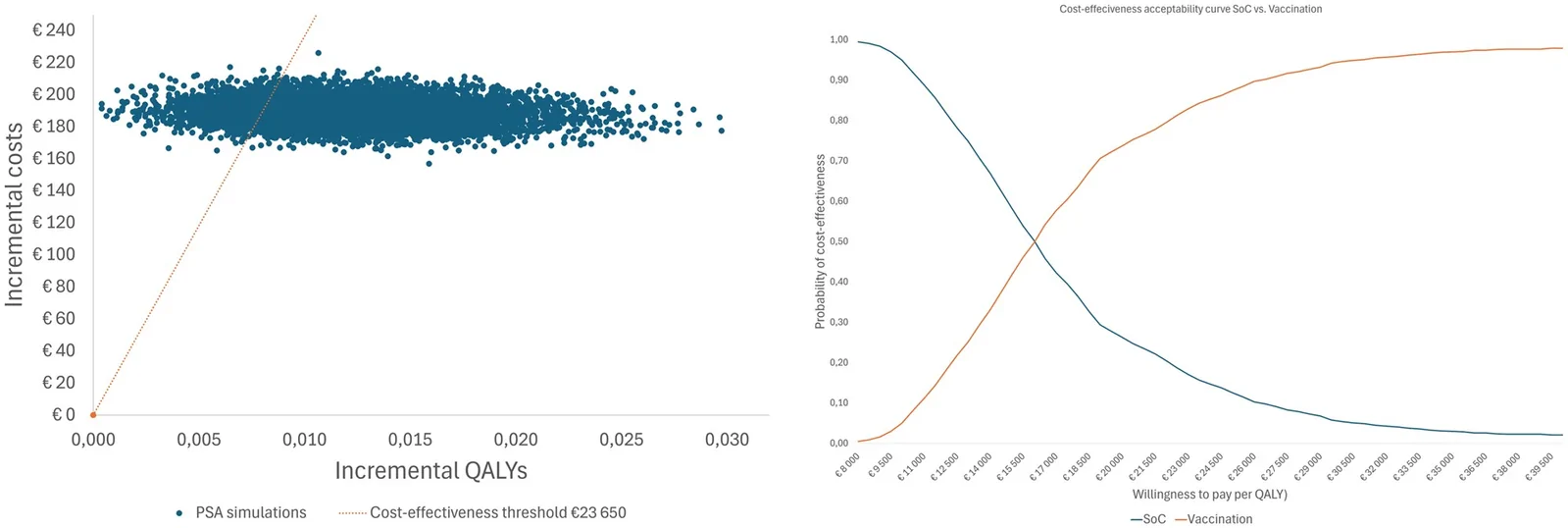
Results from the probabilistic sensitivity analysis. Cost-effectiveness scatter plot, displaying the range of possible ICERs and cost-effectiveness acceptability curve showing probability of cost-effectiveness at different thresholds

In probabilistic sensitivity analysis, vaccination generated better health and higher costs, with a probability of 98% of being considered cost-effective when compared with the assumed cost-effectiveness threshold of EUR 23 650 per QALY, while the CEAC show the probability of cost-effectiveness over a range of different cost-effectiveness thresholds (Fig. 4).

## Discussion

This study appears to provide the first cost-effectiveness analysis of the adjuvanted recombinant zoster vaccine in the context of inflammatory joint disease, addressing a crucial evidence gap. We found the RZV vaccine to be effective in reducing the incidence of HZ and PHN, leading to better health but also higher costs. In the base-case scenario, vaccination produced an ICER of EUR 11 205 per QALY, which was deemed cost-effective.

Although no other analyses are currently available for patients with RA, previous studies evaluating the cost-effectiveness of HZ vaccination in various groups of immunocompromised populations have been performed. A wide range of results have been reported, and conclusions seem to be dependent on the epidemiological factors. Salem *et al.* estimated that vaccination could be cost saving for renal transplant patients, while an ICER as high as USD 95 972 was reported for patients with Hodgkin’s lymphoma. Estimated QALY gains per person ranged from 0.002 to 0.007, depending on the patient group analysed [40].

Curran *et al.* investigated the cost-effectiveness of zoster vaccination of different groups of cancer patients and found 109 QALYs gained per 19 671 patients with hematopoietic stem cell transplant vaccinated, 506 QALYs gained per 279 100 breast cancer patient vaccinated and 17 QALYs gained per 8480 patient with Hodgkin’s lymphoma vaccinated, resulting in 0.0055, 0.0018 and 0.0020 QALYs gained per patient with the respective disease [41].

Cost-effectiveness of zoster vaccination for immunocompromised patients with hematopoietic stem-cell transplant, breast cancer, renal transplant, human immunodeficiency virus and Hodgkin lymphoma were recently investigated by George *et al.* in a Canadian setting [42]. Incremental QALY gains were not reported, but they found vaccination to be cost-effective for two groups: patients with breast cancer and patients who had undergone renal transplant.

Compared with the published estimates, our gain of 0.016 QALYs per vaccinated patient is large. One explanation for the variation across published cost-effectiveness analyses seem to be the underlying epidemiological data (notably HZ incidence and PHN risk), because higher risk populations obtain larger absolute benefits from vaccination. The tornado diagram indicated that the probability of PHN has a great impact on the results. As the conclusion on cost-effectiveness can be sensitive to the baseline risk, each group of immunocompromised patients should be evaluated separately. This underscores the need for an RA specific analysis and highlights the importance of using appropriate epidemiological inputs.

Risk of infection depends both on age and on other factors, such as immunosuppression. For this reason, many countries have separate vaccination recommendations based on age (often 65+) and other risk factors. In this analysis, we analysed patients with RA from age 50 and did not explore older age groups. We believe that in most settings, older individuals will be covered by an age specific recommendation for the general population, while a high-risk group like RA, could be cost-effective even from a younger age. As vaccination seems to be cost-effective in our base case, increasing age is likely to further improve cost-effectiveness.

In the absence of national estimates of HZ incidence in Patients with RA, we used Swedish data from two large regions, which we consider a credible alternative. The incidence of HZ in the overall population in this study is 4.2 per 1000 patient years, compared with 3.9 per 1000 in Norway (unpublished data, Norwegian Institute of Public Health). For comparison, the placebo arm of the vaccine trial recruiting from 18 countries reported 9.1 per 1000 [34]. While the HZ risk in patients with RA receiving treatment with TNF or JAK inhibitors is generally reported to be 2–3 times that of the general population, Södergren report an incidence of HZ that is 3.8 times higher for the age group 50–64 and 2.97 higher for the 65 + [20]. Generally, vaccination will be more beneficial the higher the risk of infection, hence the cost-effectiveness of RZV vaccination of patients with RA will likely vary across the relevant treatments, i.e. across methotrexate, biologic- and targeted synthetic disease modifying drugs. A recent EULAR abstract based on nationwide data from Sweden indicates that treatment with biologic disease modifying drugs doubles the risk of HZ, while JAK inhibitors are associated with a four-fold increase [43]. Data like this will facilitate future cost-effectiveness analyses that may account for treatment heterogeneity more explicitly.

PHN is challenging to track in administrative data due to varying definitions and coding practices. We assumed a PHN probability of 25% based on a prospective cohort of patients with RA [22]. Published estimates range between 5% and 42%, depending on population and study design [19, 44]. A wide range of PHN probabilities has also been used in published cost-effectiveness studies [29]. Estimates from the clinical trials on vaccine effectiveness are between 12% and 22% depending on the characteristics of the included populations [45], with the highest number reported in a trial of immunocompromised patients who had undergone autologous haematopoietic cell transplant.

In the base case analysis, we used effectiveness estimates specific to Patients with RA from clinical trials. These estimates reflect a situation where patients were instructed not to take immunosuppressing treatment in the weeks before vaccination. Recent findings from pneumococcal vaccination of patients with RA, indicate that delaying methotrexate before vaccination can improve vaccine responses [46], but postponing treatment may however not be a feasible alternative for all patients. In these situations, results based on effectiveness estimates from real-world data may be more accurate [8].

Our study is subject to several limitations. Utility estimates for HZ and PHN were derived from a study conducted in China [23]. Their direct applicability to a Norwegian population remains uncertain, although utility estimates are generally found to be transferrable between countries [47]. The study employed rigorous methodology and provided more detailed utility data on PHN than other sources. An alternative could have been to use the EQ-5D estimates reported from the clinical trials [48], but these report over a shorter time period (up to 4r weeks) and only report numbers for acute zoster infection and not PHN.

We did not include treatment-specific health-related quality of life weights for vaccinated and unvaccinated individuals who experienced an outbreak of HZ. Data from Giannelos *et al.* suggest that vaccination may reduce disease severity, thereby improving the utility of affected patients [49]. Excluding this may have led us to underestimate the overall benefit of vaccination. However, due to the low incidence of breakthrough cases, we consider this effect unlikely to significantly alter our conclusions. Additionally, including this effect would only further strengthen the conclusion that vaccination is cost-effective.

The vaccine is relatively new, and long-term follow-up data in immunocompromised populations are limited. Our assumption regarding vaccine waning (2.5% per year) was derived from a linear regression of the first 10 years of effectiveness data from the ZOE-LTFU study [25], which shows an initial decline followed by a plateau (years 6–8) and a modest drop thereafter. Because a linear approach does not capture the non-linear waning pattern observed in immunocompetent adults, it may overestimate waning in the general population while potentially underestimating waning in immunosuppressed patients such as those with RA.

We did not include any adverse effects of vaccination in the analysis. Vaccination will often lead to an injection site reaction; however, this effect is usually short, and the effect would be negligible when measured in QALYs. These reactions seldom require medical attention, hence also has a low impact on costs. There have been some concerns that vaccination may trigger flares in patients with RA. A recent phase four safety study included 1192 patients, with a primary outcome of flare occurrence. They found no effect of HZV vaccination on disease activity [10].

The structure of our model is consistent with previous economic evaluations, supporting its suitability for assessing HZ vaccination in this population [29]. However, we made several simplifying assumptions. The model did not account for rare but serious complications of HZ, such as disseminated zoster, central nervous system involvement and vision loss following HZ ophthalmicus. Although uncommon, these complications are more common in immunocompromised individuals and can lead to substantial reductions in quality of life and increased long-term healthcare costs [50]. Omitting such complications may therefore underestimate the vaccine’s overall health and economic benefits.

Dynamic transmission models are typically employed to model the effects of vaccination because they capture herd effects, i.e. changes in infection risk as coverage increases. Our study utilized a static model because HZ is a reactivation disease and is not transmitted in the same way as many other vaccine-preventable infections. Given the lack of meaningful transmittivity, and the fact that our study population represents only a small subgroup of the Norwegian population, herd effects were deemed negligible. Thus, we believe that the simplification of using a static model is unlikely to significantly impact our results.

We applied a cost-effectiveness threshold as recommended for Norway. Cost-effectiveness thresholds vary across the globe [18]. The applied threshold is low compared with thresholds applied in many other countries (e.g. in a US setting, one would often apply $100 000–150 000, while the UK recently increased its threshold to £2500–35 000). The implication is that vaccination would be considered more cost-effective if the threshold is higher. The included CEAC does however show likelihood of cost-effectiveness over a range of thresholds.

Any health economic model is a simplified representation of reality. It is practically impossible, and often unnecessary, to incorporate every real-world detail. Simple models are more straightforward to interpret and implement, yet they may omit significant factors. Conversely, more complex models can closely align with reality, but they risk reduced transparency and increased potential for error. Ideally, a model should encapsulate the essential features of the disease process without introducing unnecessary complexity.

More precise estimates of HZ and PHN incidence in rheumatoid arthritis patients would help enhance the accuracy of future models, as these parameters exhibit considerable uncertainty and are key drivers of cost-effectiveness results. Long-term follow-up data on the effectiveness and waning of the vaccine in immunocompromised populations are also required. Additionally, further investigation into the quality of life and healthcare utilization for rheumatoid arthritis patients with HZ and postherpetic neuralgia would strengthen the validity of estimates in upcoming models.

## Data Availability

No new patient data were generated or analysed in support of this research. Model files used for this analysis will be shared on reasonable request to the corresponding author.
